# Natural Course of Myoclonus‐Dystonia in Adulthood: Stable Motor Signs But Increased Psychiatry

**DOI:** 10.1002/mds.28033

**Published:** 2020-03-25

**Authors:** Elze R. Timmers, Kathryn J. Peall, Joke M. Dijk, Rodi Zutt, Cees C. Tijssen, Bruno Bergmans, Elisabeth M. Foncke, Marina A.J. Tijssen

**Affiliations:** ^1^ Department of Neurology University Medical Center Groningen, University of Groningen Groningen the Netherlands; ^2^ Neuroscience and Mental Health Research Institute Division of Psychological Medicine and Clinical Neurosciences, Cardiff University Cardiff Wales United Kingdom; ^3^ Department of Neurology, Amsterdam UMC University of Amsterdam Amsterdam the Netherlands; ^4^ Department of Neurology Elisabeth‐TweeSteden Ziekenhuis Tilburg the Netherlands; ^5^ Department of Neurology A.Z. Sint‐Jan Brugge‐Oostende AV Bruges Belgium; ^6^ Department of Neurology Ghent University Hospital Ghent Belgium

**Keywords:** follow‐up, myoclonus‐dystonia, nonmotor symptoms

Myoclonus‐dystonia (M‐D) is a rare hyperkinetic movement disorder characterized by upper body–predominant myoclonus and dystonia.^1^ A large proportion of cases are caused by autosomal‐dominant inherited mutations in the *SGCE* gene. In addition to the motor manifestations, psychiatric disorders are frequently reported.^2^ Several studies have suggested that they may form a primary component of the M‐D phenotype.^3^, ^4^ This study represents the first long‐term follow‐up study of both motor and psychiatric symptomatology in adults with M‐D (*SGCE* mutation), providing further insights into the natural history of M‐D and enabling more prognostic information.

## Methods

Manifesting adult carriers with a mutation in the *SGCE* gene were included, whose baseline data were collected in the Netherlands, Belgium, and the United Kingdom and reported previously.^2^, ^3^, ^5^

Regarding motor signs, both at baseline and follow‐up, Burk Fahn Marsden Dystonia Rating Scale (BFMDRS) and Unified Myoclonus Rating scale (UMRS) were used to objectively assess motor sign severity. Psychiatric comorbidity and quality of life were evaluated using the same (or highly comparable) questionnaires at baseline and follow‐up.

## Results

Of the 63 adult M‐D patients recruited in the original studies, 27 patients were able to participate in this follow‐up assessment. An overview of body distribution and severity scales of motor signs at baseline and follow‐up examination can be found in Table 1. No age‐distinctive pattern in the scores was observed. See Table 1 for the prevalence of psychiatric disorders and scores of severity scales. No associations between changes in motor symptoms and psychiatric symptom severity, quality of life, and demographic information were found.

**Table 1:** Demographics, motor symptoms, and psychiatric comorbidities

Total number of patients	27	
Sex M/F	11/16	
Age at onset dystonia (range)	8 (1–40)	
Age at onset myoclonus (range)	7 (1–17)	
Oral medication for motor symptoms	6 (22%)	
Antidepressant		
SSRI	4 (13%)	
SNRI	2 (7%)	
TCA	1 (3%)	
Botulinum neurotoxin injections	3 (11%)	

Motor symptoms	Baseline examination	Follow‐up examination	*P*
Age (SD)	44.6 (14.3)	55.2 (14.4)	
Number of patients with:			
Dystonia	23 (85%)	25 (93%)	0.625^a^
Myoclonus	22 (81%)	24 (89%)	0.500^a^
Body distribution			
Dystonia (n = 21)			
— Upper limbs	5 (24%)	18 (86%)	**< 0.001** a
— Lower limbs	2 (10%)	5 (24%)	0.250^a^
— Neck	18 (86%)	19 (90%)	1.000^a^
— Trunk	5 (24%)	8 (38%)	0.453^a^
Myoclonus (n = 22)			
— Upper limbs	20 (91%)	20 (91%)	1.000^a^
— Lower limbs	1 (5%)	7 (32%)	0.070^a^
— Neck	8 (36%)	16 (73%)	**0.008** a
— Trunk	6 (27%)	15 (68%)	**0.012** a
All patients			
BFMDRS, n = 24 (range)	3.5 (0–20)	6.6 (0–17.5)	0.203^b^
UMRS, n = 24 (range)	2.3 (0–12)	3.7 (0–12)	0.140^b^
Patients with mutation inherited via paternal line			
BFMDRS, n = 19 (range)	4.0 (0–20)	7.0 (1–17.5)	0.198^b^
UMRS, n = 19, (range)	5.2 (0–12)	5.4 (0–12)	0.260^b^

	Baseline examination	Follow‐up examination	
Psychiatry	n = 26	n = 6	*P* a
Any psychiatric disorder	16 (62%)	20 (77%)	0.219
Depression	7 (27%)	13 (50%)	0.070
Panic disorder	6 (23%)	12 (46%)	**0.031**
Social phobia	6 (23%)	4 (15%)	0.688
OCD	3 (12%)	6 (23%)	0.453
Alcohol dependence	3 (12%)	3 (12%)	1.000
GAD	3 (12%)	3 (12%)	1.000
Specific phobia	3 (14%)^3^	5 (23%)^3^	0.688
Agoraphobia	4 (33%)^4^	6 (50%)^4^	0.500
Hypomania	1 (10%)^6^	1 (10%)^6^	1.000
Psychosis	1 (10%)^6^	2 (20%)^6^	1.000

	n = 22	n = 22	*P* b
YBOCS (range)	0.0 (0–14)	0.0 (0–12)	0.634
BDI (range)	5.0 (0–21)	7.5 (0–30)	**0.038**
BAI (range)	5.0 (0–24)	7.5 (0–45)	0.070

	n = 13	n = 13	*P* c
QoL PC (SD)	44.8 (8.8)	45.2 (6.9)	0.875
QoL MC (SD)	38.8 (13.6)	43.0 (9.2)	0.193

The following statistical tests were used:

a McNemar test.

b Wilcoxon signed rank test.

c Paired‐sample *t* test: 1n = 9; ^2^n = 5; ^3^n = 22; ^4^n = 12; ^5^n = 7; ^6^n = 10.

SSRI, selective serotonin reuptake inhibitor; SNRI, selective serotonin and noradrenalin reuptake inhibitor; TCA, tricyclic antidepressant; BFMDRS, Burke Fahn Marsden Dystonia Rating Scale; UMRS, Unified Myoclonus Rating Scale; OCD, obsessive compulsive disorder; GAD, generalized anxiety disorder; YBOCS, Yale Brown Obsessive Compulsive Scale; BDI, Beck Depression Inventory; BAI, Beck Anxiety Inventory; QoL PC, physical component of quality of life; QoL MC, mental component of quality of life.

## Discussion

This is the first systematic long‐term follow‐up study of both motor and psychiatric manifestations in adult M‐D patients. Despite the percentage of patients retested (27 of 63), the rarity of M‐D makes our results valuable. Results of this study showed that in adulthood the course of dystonia and myoclonus is static and the prevalence of psychiatric comorbidities remains high. Specific psychiatric disorders, notably panic disorder and depression, became even more prevalent over time.

It appears that in adulthood severity of motor manifestations is relatively stable, but distribution lightly changed. At follow‐up examination significantly more patients had dystonia in the upper limbs and more patients had myoclonus in the neck and trunk compared with baseline. This is consistent with previous findings.^6^

Comparable to the literature, psychiatric comorbidity was highly prevalent in our cohort. The prevalence of panic disorder doubled at follow‐up compared with baseline and was accompanied by an increased score on the anxiety severity scale. Similar, but not statistically significant, findings were detected for depressive disorder. It is unlikely that our findings are because of an increase in age, as the prevalence of panic disorder and depression in the general population tends to decrease in the age group of our cohort.^7^

The relatively stable course of motor manifestations is in contrast with the increased prevalence of psychiatric comorbidity. Results highlight the need for more awareness and adequate treatment for psychiatric disorders in M‐D patients. Simultaneously, adult patients can be reassured that their motor functioning will not deteriorate.

## Author Roles

1) Research project: A. Conception, B. Organization, C. Execution; 2) Statistical Analysis: A. Design, B. Execution, C. Review and Critique; 3) Manuscript: A. Writing of the first draft, B. Review and Critique.

E.R.T.: 1A, 1B, 1C, 2A, 2B, 3A.K.J.P.: 1B, 1C, 2A, 2C, 3A, 3B.J.M.D.: 1C, 2C, 3B.R.Z.: 1C, 2C, 3B.C.C.T.: 1B, 1C, 2C, 3B.B.B.: 1B, 2C, 3B.E.M.F.: 1C, 2C, 3B.M.A.J.T.: 1A, 1B, 1C, 2C, 3A, 3B.

## Financial Disclosures of all authors (for the preceding 12 months)

M.A.J.T. and E.R.T. received a grant from the Dystonia Medical Foundation for conducting this study. M.A.J.T. reports grants from the European Fund for Regional Development (01492947) and the province of Friesland, Stichting Wetenschapsfonds Dystonie Vereniging, Fonds Psychische Gezondheid, Phelps Stichting, from Ipsen & Allergan Farmaceutics, Merz, and Actelion. All other authors report no financial disclosures.
